# Biodegradable Nanofiber Membranes for Air–Liquid Interface Culture: Advancing Airway *In Vitro* Models

**DOI:** 10.1021/acsomega.5c03198

**Published:** 2025-08-21

**Authors:** Sema Tuncer, Secil Subasi Can, Hayriye Akel Bilgic, Busra Kilic, Gulcin Gunal, Halil Murat Aydin, Cagatay Karaaslan

**Affiliations:** a Institute of Science and Centre of Bioengineering, Bioengineering Division, 37515 Hacettepe University, Cankaya, Ankara 06800, Türkiye; b Faculty of Science, Department of Biology, Molecular Biology Section, 37515 Hacettepe University, Cankaya, Ankara 06800, Türkiye; c Faculty of Medicine, Department of Plastic Surgery, 37502 Akdeniz University, Campus, Konyaaltı, Antalya 07070, Türkiye

## Abstract

Chronic airway diseases represent a significant global health challenge, and reliable *in vitro* model systems are essential for elucidating the molecular mechanisms underlying these conditions. Although air–liquid interface (ALI) culture systems are among the most effective models for studying airway epithelial cells under physiological conditions, the nonbiodegradable membranes commonly used in current systems present certain limitations. In the present study, biodegradable poly­(l-lactic acid) (PLLA) and poly­(ε-caprolactone) (PCL) nanofiber membranes were fabricated using the electrospinning technique, and novel transwell membrane systems were developed. Optimization studies revealed that the nanofiber diameters ranged between 50 and 275 nm, forming a structure closely resembling the native lung extracellular matrix. Degradation analyses indicated that PLLA and PCL membranes remained structurally stable for up to six months, making them suitable for long-term *in vitro* airway modeling applications. Attenuated total reflectance–Fourier transform infrared (ATR-FTIR) spectroscopy confirmed the chemical stability of the membranes. Additionally, cell culture assays demonstrated high cell viability and strong cellular adhesion. Immunocytochemical analysis revealed β-tubulin expression in bronchial epithelial cells differentiated on the membranes, indicating successful epithelial maturation. These findings suggest that biodegradable membranes provide a promising platform for *in vitro* airway modeling. Furthermore, the use of biodegradable membranes is expected to address a critical need by accurately mimicking the tracheal and bronchial architecture as submucosal tissue analogues, thereby advancing the development of preclinical airway tissue graft constructs.

## Graphical abstract

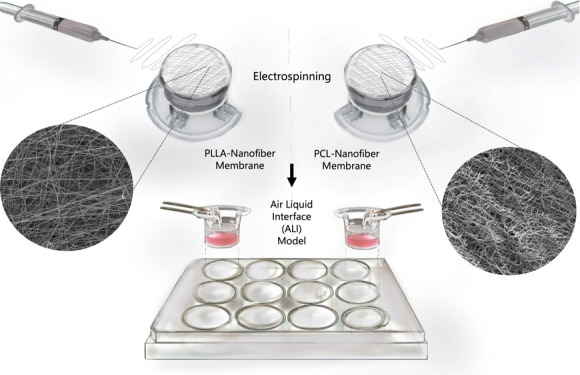

## 1 Introduction

Chronic airway diseases are heterogeneous and complex conditions resulting from the interplay between genetic predispositions and environmental exposures. The intricate three-dimensional (3D) architecture of the lung presents a major challenge for accurately replicating pulmonary tissues *in vitro*. Understanding the pathophysiology of these diseases requires advanced cell culture models that can reflect the anatomical and physiological diversity of lung cell types. Tissue engineering has emerged as a crucial discipline for investigating the molecular complexity of chronic airway diseases and for developing effective therapeutic strategies. In recent years, significant advances have been made in lung tissue engineering, particularly with the development of 3D cell culture models, including scaffold-based systems, air–liquid interface (ALI) cultures, microfluidic devices, spheroids, and lung tissue explants.^1,2^ Among these, the ALI culture system—employing transwell membrane inserts—has garnered considerable attention due to its capacity to mimic key aspects of pulmonary physiology in an *in vitro* environment.^3,4^ In this approach, cells are initially seeded onto a permeable membrane and nourished from both apical and basal compartments. During the differentiation phase, the apical medium is removed, allowing the cells to be exposed to air at the top while receiving nutrients exclusively from the basal side. This configuration enables the partial recreation of lung physiology in 3D *in vitro* settings by mimicking the air exposure characteristic of the airway epithelium.^5^

In 3D cell culture systems, scaffolds play a critical role in supporting cell adhesion, growth, and migration. These scaffold-based cultures rely on a structural framework that provides the necessary physical support, enabling cells to aggregate, proliferate, and migrate in a spatially organized manner. Scaffold-based models encompass a range of tissue engineering strategies, including the use of synthetic or natural membranes, decellularized extracellular matrices, hydrogels, and 3D-printed structures.^1^ Scaffolds produced through these tissue engineering strategies are expected to exhibit properties that closely mimic the native lung environment, including appropriate mechanical strength, surface characteristics, and elasticity. Such biomimetic features are essential for the development of tissue grafts that support lung regeneration through physiologically relevant designs and models.

Tissue engineering approaches frequently utilize both synthetic and natural polymers, as well as hybrid structures combining the two. Among the most commonly used materials in lung tissue engineering are synthetic polymers such as polylactic acid (PLA), polyglycolic acid (PGA), poly­(lactic-*co*-glycolic acid) (PLGA), and poly­(ε-caprolactone) (PCL), as well as natural biopolymers including collagen, elastin, silk fibroin, and chitosan.^6^ Among membrane-based scaffold fabrication techniques, electrospinning is widely recognized as an effective and cost-efficient method for producing tissue scaffolds suitable for cell culture applications. Electrospun membranes offer great potential for biomedical use, owing to their tunable physical and chemical properties, along with a high surface area-to-volume ratio. The fibrous architecture of electrospun scaffolds closely resembles the native extracellular matrix (ECM), providing a temporary yet functional microenvironment that supports cellular adhesion, proliferation, and tissue regeneration.^7^

Creating a microenvironment that closely mimics native tissue architecture through 3D coculture systems remains a central goal of *in vitro* research. Accurately replicating cell–cell interactions within these 3D models is crucial for understanding cellular functions, mechanisms, and metabolic processes. However, the compatibility of commercially available platforms with 3D culture setups remains limited. Moreover, commonly used polymer-based membranes in transwell systems—such as polyethylene glycol (PEG), polyethylene terephthalate (PET), and polytetrafluoroethylene (PTFE)—are nonbiodegradable and often exhibit poor biocompatibility, characterized by minimal cell adhesion. Biocompatibility refers to the capacity of tissue engineering scaffolds or matrices to support appropriate cellular activities, including the facilitation of molecular and mechanical signaling pathways that optimize tissue regeneration without inducing adverse local or systemic responses in the host.^6,8^ Therefore, although microporous membrane structures are employed in commercially available transwell inserts, these products remain unsuitable for applications as lung tissue grafts.

Electrospun nanofiber membranes have emerged as promising scaffolds for modeling airway epithelial barriers due to their high surface area, porosity, and structural similarity to native extracellular matrices. Among the various biodegradable polymers, poly­(l-lactic acid) (PLLA) and poly­(ε-caprolactone) (PCL) have been widely studied for their biocompatibility and tunable mechanical properties. However, despite their frequent use in tissue engineering, few studies have directly compared their performance under standardized ALI culture conditions, particularly in the context of long-term epithelial barrier formation.

Existing research has typically focused on a single polymer system and short-term endpoints such as cell adhesion or morphology. Quantitative assessments of epithelial barrier integrity—such as transepithelial electrical resistance (TEER) measurements—remain limited, especially for extended culture durations. Furthermore, the influence of membrane-specific factors such as thickness, fiber morphology, and mechanical properties on epithelial maturation and function is not yet fully understood.^9,10^

To address these gaps, the present study offers a side-by-side comparison of electrospun PLLA and PCL membranes in a 21-day ALI culture model. By evaluating structural, mechanical, and functional parameters, this work aims to inform the rational selection and optimization of biodegradable nanofibrous membranes for respiratory tissue engineering and epithelial barrier modeling.

To overcome the limitations of existing pulmonary airway cell culture systems and introduce novel approaches, we developed a biodegradable ALI culture system aimed at facilitating epithelial tissue graft formation and enabling long-term airway modeling. For this purpose, two FDA-approved polymers, PLLA and PCL, were fabricated via electrospinning to produce nanofibrous network membranes, which were subsequently integrated into the ALI culture system. The resulting membranes were thoroughly characterized with respect to their morphological, structural, mechanical, and cytotoxic properties.

In this study, we propose that PLLA and PCL membranes can overcome the aforementioned limitations and serve as robust alternatives to the conventional nonbiodegradable membranes used in traditional ALI culture systems.

## 2 Materials and Methods

### 2.1 Fabrication of Nanofiber Membranes

The fabrication process of PLLA and PCL nanofiber membranes was optimized based on the parameters listed in Table 1. Briefly, a 4% (w/v) PLLA solution (Purac-PL24 MW: 290,000–300,000 g/mol, Corbion, Netherlands) was prepared by dissolving the polymer in a 1:1 (v/v) mixture of dichloromethane (DCM, Sigma-Aldrich, ≥ 99.8%) and dimethylformamide (DMF, Sigma-Aldrich, ≥ 99.8%) under magnetic stirring at 50 °C for 48 h. Similarly, a 17% (w/v) PCL solution was prepared by dissolving the polymer (Sigma-Aldrich, USA) in a 1:1 (v/v) mixture of tetrahydrofuran (THF, Merck) and DMF, stirred at room temperature until fully dissolved.

**Table 1:** Electrospinning Parameters Used for the Fabrication of PLLA and PCL Nanofiber Membranes

	**PLLA**	**PCL**
concentration (w/v)	4%	17.5%
voltage (kV)	17	17
distance (cm)	10	7.5
feed rate (mL/h)	1	1.5
needle diameter (G)	26	26

Electrospinning of PLLA nanofibers was carried out at a flow rate of 1 mL/hour under an applied voltage of 17 kV, with a 10 cm distance between the 26-gauge syringe needle and the collector. For PCL nanofibers, a higher flow rate of 1.5 mL/h was used, maintaining the same voltage (17 kV), but with a shorter electrode distance of 7.5 cm. In both cases, fibers were collected on a grounded aluminum foil substrate for 2 h.^11–13^

### 2.2 Characterization of Nanofiber Membranes

#### 2.2.1 Scanning Electron Microscopy (SEM) Imaging

To evaluate the morphology, diameter, and distribution of electrospun PLLA and PCL fibers, scanning electron microscopy (SEM) analysis was conducted using a TESCAN GAIA3 system (Czechia) at the Hacettepe University Advanced Technologies Application and Research Center (HUNITEK). For SEM imaging, membrane samples measuring 1 cm^2^ were sputter-coated with a 4 nm layer of gold/palladium (Au/Pd) using a Leica EM ACE600 coater (Germany). The coated samples were mounted on conductive carbon tape (stubs), and images were acquired at various magnifications. Fiber diameter measurements were performed on randomly selected regions using ImageJ software (version IJ 1.46r Fifi win-64), and results were expressed as mean diameter ± standard deviation.^14^

#### 2.2.2 Pore Size Analysis by Capillary Flow Porometry

Pore size and distribution of the electrospun membranes were measured using a capillary flow porometer (Quantachrome 3G, USA). PLLA and PCL membranes were cut into 18 mm diameter discs to fit the sample chamber and then placed into the porometer for analysis.^15,16^

#### 2.2.3 Determination of Membrane Thickness

To determine the thickness of the electrospun membranes, the samples were carefully detached from the collector plate and cut into 3 cm × 3 cm pieces. Thickness measurements were performed using a digital caliper (Insize, China; measurement range: 0–150 mm/0–6 in.; accuracy: ± 0.03 mm). For each of the ten samples, three different points were measured, and the results were reported as mean ± standard deviation.^17^

#### 2.2.4 Attenuated Total Reflectance–Fourier Transform Infrared Spectroscopy (ATR-FTIR)

The chemical structure of the electrospun PLLA and PCL membranes, as well as potential impurities arising from the fabrication process, was analyzed using an ATR-FTIR spectrophotometer (Thermo Scientific Nicolet iS10, USA).^14,18^

#### 2.2.5 Mechanical Test

Tensile testing of the electrospun PLLA and PCL membranes was performed on samples measuring 1 cm × 1 cm using a universal testing machine (Zwick/Roell Z250, Germany) with a 50 N load capacity (Figure S1). Three samples from each group were tested at a crosshead speed of 10 mm/min under ambient conditions. The elastic modulus was calculated from the linear region of the stress–strain curve, specifically between 2% and 20% strain. Results were reported as mean ± standard deviation.^19,20^

#### 2.2.6 Biodegradability of Nanofiber Membranes

To assess *in vitro* biodegradability, PLLA and PCL membranes were cut into 1 cm × 1 cm squares. The initial dry weight (*D*_0_) of each sample was measured using a precision analytical balance. Samples were then sterilized by immersion in 70% (v/v) ethanol for 2 h, followed by three washes with phosphate-buffered saline (PBS). After sterilization, the membranes were incubated in Ringer’s solution at 37 °C for a total of six months.

At monthly intervals (from the first to the sixth month), the samples were removed, dried, and reweighed to determine their final dry weight (*D*_f_). The weight loss was calculated using the following equation:

$$\mathrm{weight}\;\mathrm{change}\left(\mathrm{mg}\right)=\left(D_{0}-D_{f}\right)$$ 1

To assess enzymatic degradation behavior, PLLA membranes were cut into 1 cm × 1 cm squares and incubated in 1 mL of proteinase K solution (2 mg/mL) at 37 °C for 28 days in a shaking incubator. Membranes were retrieved at predetermined time points (days 7, 14, 21, and 28), gently rinsed with distilled water, dried, and weighed to determine the residual dry mass. The percentage of degradation was calculated by comparing the remaining weight with the initial dry weight. The experiment aimed to simulate enzymatic degradation under physiologically relevant conditions and evaluate the stability of the membranes in the presence of proteolytic activity.

#### 2.2.7 Cytotoxicity Analysis

The *in vitro* cytotoxicity of electrospun PLLA and PCL membranes was assessed by ISO 10993-5 and ISO 10993-12 standard protocols.^21,22^ Cytotoxicity analyses of the fabricated membranes were conducted using L929 mouse fibroblast cells (NCTC clone 929, ATCC). Detailed experimental procedures are provided in the Supporting Information.^18^

### 2.3 Cell Culture Studies

#### 2.3.1 Cell Proliferation, Histology, and SEM Imaging of BEAS-2B Cells on PLLA and PCL Membranes

##### 2.3.1.1 Cell Proliferation

Cell proliferation on PLLA and PCL membranes was assessed by quantifying the increase in DNA content (ng/mL) using the CyQUANT Cell Proliferation Assay Kit (Thermo Fisher Scientific, USA), following the manufacturer’s protocol.^23^ For this purpose, PLLA and PCL membranes were cut into 6 mm diameter disks compatible with 96-well plates. The membranes were secured using Kwik-Sil (World Precision Instruments, Sarasota, FL), a biocompatible silicone adhesive widely used in biomedical applications for its low cytotoxicity and reliable fixation. The membranes were sterilized by immersion in 70% (v/v) ethanol (Merck, ≥ 99.8%, Germany) for 2 h, followed by washing with phosphate-buffered saline (PBS) (Gibco, Thermo Fisher Scientific, USA) and air drying. To enhance cell adhesion, 50 μL of 10% (v/v) fetal bovine serum (FBS) (Gibco, Cat. No. 26140079) was added to each well, and the membranes were incubated overnight at 37 °C in a humidified atmosphere containing 5% CO_2_.^24,25^ Bronchial epithelial cells (BEAS-2B) (ATCC CRL-9609, USA) were seeded at a density of 1.0 × 10^5^ cells per well. Cell proliferation was assessed on days 1, 2, 3, and 4 of the experiment.^26–28^

##### 2.3.1.2 Hematoxylin and Eosin (H&E) Staining

To assess the adhesion of BEAS-2B cells to the membrane surfaces, PLLA and PCL membranes were first fixed in 4% (w/v) paraformaldehyde solution and subsequently immersed in 30% (w/v) sucrose solution (in PBS) (Sigma-Aldrich, Germany) overnight. The samples were then embedded in 100% OCT compound (Tissue-Tek OCT Compound, Sakura Finetek, USA), and 5 μm-thick sections were prepared using a cryostat (Leica CM1950, Leica Biosystems, Germany). Sections were stained with Hematoxylin and Eosin (H&E) using a commercial staining kit (ScyTek Laboratories, USA) following the manufacturer’s protocol.^29^

##### 2.3.1.3 SEM Imaging

Morphological analysis of BEAS-2B cells cultured on the membranes was performed using SEM. The membranes were initially fixed in 2.5% (w/v) glutaraldehyde (Sigma-Aldrich, ≥ 25% in H_2_O, Merck, Germany) for 1 h, followed by dehydration through a graded ethanol series (30%, 50%, 70%, 80%, 90%, and 100%) (Ethanol, Merck, ≥ 99.8%, Germany). Further dehydration was conducted using hexamethyldisilazane (HMDS) (Sigma-Aldrich, ≥ 99%, USA) for 10 min. Subsequently, samples were air-dried overnight in a fume hood and sputter-coated with a gold–palladium (Au–Pd) layer before imaging.^30^

##### 2.3.1.4 Immunofluorescence Staining and Trans-Epithelial Electrical Resistance (TEER)

β-Tubulin expression was assessed to evaluate the differentiation status of bronchial epithelial cells after 21 days of culture. Briefly, cells were fixed with 4% paraformaldehyde (w/v) (Sigma-Aldrich, ≥ 95%, Germany) and permeabilized using 0.2% Triton X-100 (Sigma-Aldrich, ≥ 98%, Germany) in phosphate-buffered saline (PBS, pH 7.4) (Gibco, Thermo Fisher Scientific, USA).^31,32^ Primary antibody against β-tubulin (ab15568, Abcam, UK) was diluted 1:50 in 1% (w/v) bovine serum albumin (BSA) (Sigma-Aldrich, ≥ 98%, Germany) and applied to the membranes, followed by incubation for 2 h at room temperature. Cell nuclei were counterstained with DAPI (1 μg/mL) (D9542, Sigma-Aldrich, Germany) using Fluoromount-G mounting medium (Thermo Fisher Scientific, USA). Membranes were then covered, and images were captured from at least five random fields per sample using an EVOS Cell Imaging Station (Thermo Fisher Scientific, USA).

TEER measurements were conducted using an epithelial voltohmmeter (Millicell ERS-2, Merck, Germany). All measurements were performed in triplicate, following the same protocol across all experimental groups. Detailed experimental procedures are provided in the Supporting Information.^33,34^

### 2.4 Statistical Analysis

All statistical analyses were performed using GraphPad Prism 9 software (GraphPad Software Inc., San Diego, CA, USA). Data are presented as mean ± standard deviation (SD) from repeated measurements. To determine statistical significance among multiple groups, one-way ANOVA followed by a Bonferroni post hoc test was applied. A *p*-value < 0.05 was considered statistically significant. Significance levels are indicated as follows: ns (not significant, *p* > 0.05), *p* < 0.05 (*), *p* < 0.01 (**), *p* < 0.001 (***), and *p* < 0.0001 (****).

## 3 Results and Discussion

Tissue engineering plays a pivotal role in biomedical research by offering innovative strategies for the repair and regeneration of damaged tissues and organs. The design of scaffolds that closely mimic the native tissue microenvironment is particularly critical in lung tissue engineering, given the anatomical and physiological complexity of the respiratory system. Biomimetic scaffolds, which emulate the architecture and biochemical composition of the native ECM, have shown great promise in addressing these challenges. Such scaffolds support essential cellular processes, including cell adhesion, proliferation, and differentiation, thereby facilitating effective tissue regeneration.^35^ Among the various materials explored for scaffold fabrication, electrospun nanofiber membranes have emerged as a highly versatile platform owing to their tunable physicochemical properties and excellent biocompatibility.^36^ Producing biomimetic scaffolds for tissue-engineering applications demands meticulous optimization to elicit appropriate cellular responses while remaining compatible with the complex microenvironment of living tissues. In this study, we present a comprehensive characterization of electrospun PLLA and PCL nanofiber membranes and assess their biocompatibility, thereby elucidating their potential for *in vitro* airway modeling and related tissue-engineering applications. SEM analysis is a widely employed technique for evaluating the morphological characteristics of electrospun nanofiber membranes, providing detailed insights into fiber diameter, surface topology, porosity, and uniformity—parameters that are critical for scaffold performance in tissue engineering applications.^37^ We investigated the structural characteristics of electrospun PLLA and PCL nanofiber membranes, including fiber diameter, pore size, and overall morphology. Representative SEM images of PLLA and PCL membranes at two different magnifications are shown in Figure 1A and Figure 1B, respectively. Uniform, bead-free nanofibers were successfully produced for both PLLA and PCL using the optimized electrospinning parameters (Table 1). The distribution of fiber diameters ranged from 50 to 275 nm, as illustrated in Figure 1C,D. The mean fiber diameter was calculated to be 192 ± 49 nm for PLLA membranes and 153.33 ± 27 nm for PCL membranes.

**Figure 1:**
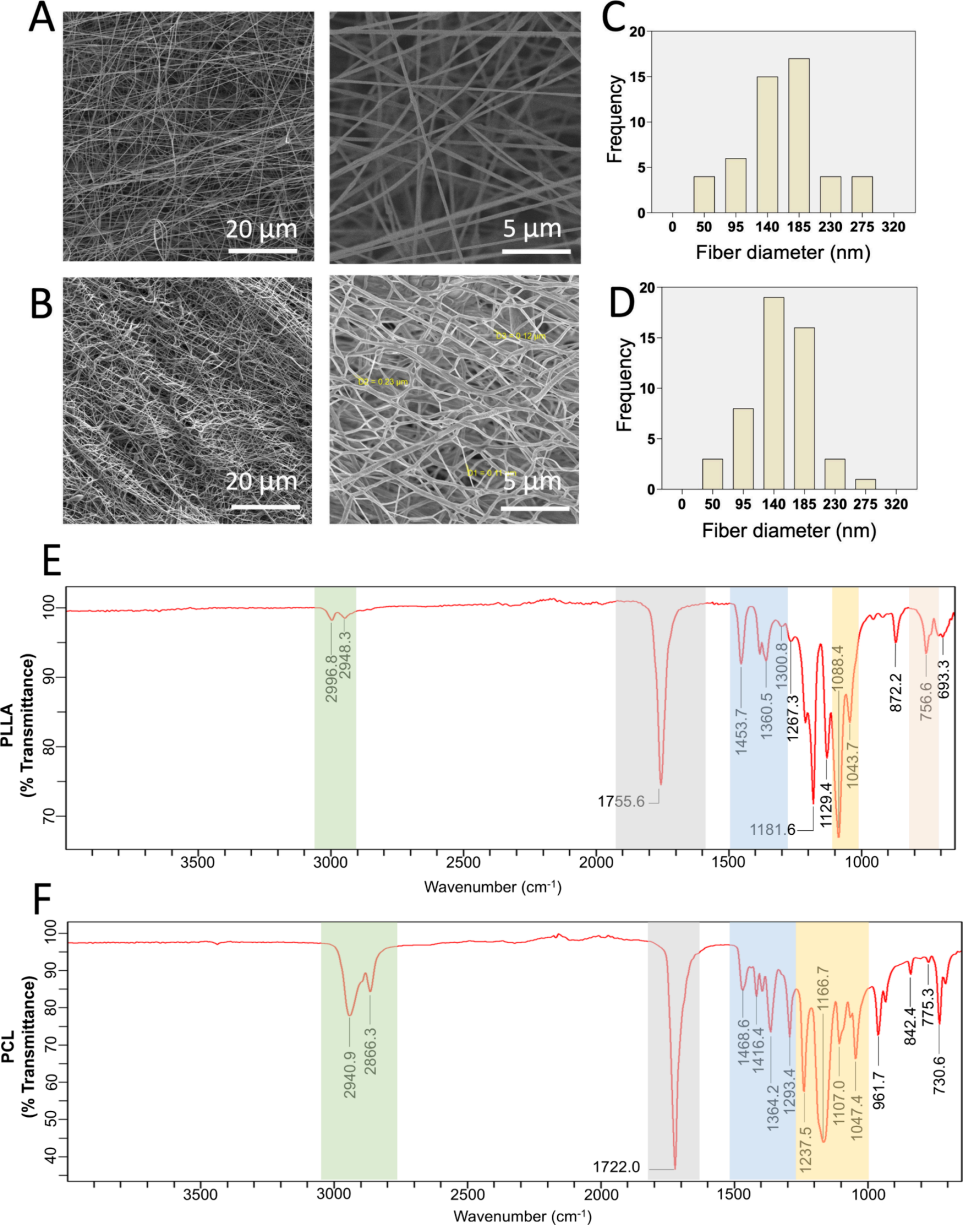
SEM images of electrospun PLLA (A) and PCL (B) nanofiber membranes at 2000× (left) and 10,000× (right) magnifications. Fiber diameter distribution histograms for PLLA (C) and PCL (D) nanofibers. FTIR spectra of PLLA (E) and PCL (F) nanofiber membranes.

Both the average fiber diameters and average pore sizes are summarized in Table 2. The average pore size of PLLA membranes ranged from 0.35 to 0.53 μm, with a mean flow pore diameter of 0.39 μm. Similarly, the pore size of PCL membranes ranged from 0.35 to 0.53 μm, with a mean flow diameter of 0.40 μm (Table 2).

**Table 2:** Average Fiber Diameters and Average Pore Sizes of PLLA and PCL Membranes

	PLLA	PCL
average fiber diameter (nm)	192 ± 49	153.33 ± 27
average pore size (μm)	0.39	0.40

SEM analysis demonstrated that the electrospun PLLA and PCL nanofiber membranes consisted of bead-free, randomly oriented fibers with diameters ranging from 50 to 275 nm. Notably, these fiber diameters closely resemble those of collagen and elastin fibers found in native lung extracellular matrices.^38^ Pore size analysis provided important insights into membrane functionality, with an average pore size of approximately 0.4 μm. Comparative studies have shown that membranes with a pore size of 0.4 μm offer optimal conditions for assessing ionic permeability and closely mimic *in vitro* primary ALI cultures as well as the *in vivo* bronchial mucosa.^39^

Figures 1E and Figure 1F present the ATR-FTIR spectra characterizing the chemical composition of PLLA and PCL membranes, respectively. In Figure 1E, corresponding to the PLLA membrane, peaks observed at 2996 cm^–1^ and 2948 cm^–1^ were attributed to CH_3_ stretching vibrations, while a prominent peak at 1755 cm^–1^ indicated the presence of C=O groups. Additional bands included asymmetric CH_3_ bending at 1457 cm^–1^, C–H stretching at 1360 cm^–1^ and 1304 cm^–1^, symmetric C–O–C bending at 1088 cm^–1^, and a C=O related peak at 755 cm^–1^. For the PCL membrane (Figure 1F), peaks at 2490 cm^–1^ and 2860 cm^–1^ corresponded to asymmetric and symmetric CH_2_ stretching, respectively, and the ester C=O bond was identified at 1722 cm^–1^. Peaks related to C–C bending and stretching appeared at 1468 cm^–1^ and 1293 cm^–1^, respectively, while asymmetric C–O–C stretching was observed at 1237 cm^–1^. Symmetric C–O–C stretching was detected at 1166 cm^–1^ and 1047 cm^–1^.

Overall, the ATR-FTIR spectra confirmed the expected chemical structures of PLLA and PCL membranes, consistent with characteristic peaks previously reported in the literature.^40–42^ These results provide comprehensive chemical characterization of the biomimetic scaffolds, reinforcing their suitability for tissue engineering applications.

Various synthetic and natural matrices have been extensively utilized in lung tissue engineering, employing tunable 2D and 3D *in vitro* ECM model systems. However, these models have thus far failed to accurately replicate the unique spatial architecture of pulmonary tissue.^43^ The ECM of the pulmonary parenchyma primarily comprises collagen, elastin, and laminin, with these structural proteins playing a critical role in determining the mechanical properties of the lung. Scaffold and membrane mechanical properties depend heavily on processing parameters, which can be tailored for specific organs and applications. Factors such as elastic modulus, fiber diameter, and fiber orientation govern these properties, with scaffold architecture serving a complementary role in optimizing overall performance.^44^

In this study, the thicknesses of PLLA and PCL membranes were measured using a digital caliper. The thicknesses of the PLLA and PCL nanofiber membranes were determined as 77 ± 12 μm and 144 ± 16 μm, respectively. Young’s moduli (MPa) of the PLLA and PCL electrospun membranes were calculated based on the stress–strain curves within the strain range of 2% to 20% (Figure S2). According to the stress–strain graphs, the elastic moduli of PLLA and PCL membranes were calculated as 6.82 ± 0.57 MPa and 5.60 ± 1.54 MPa, respectively. No statistically significant difference was observed between the elastic moduli of the PCL and PLLA membranes.

The elastic modulus of normal lung tissue has been reported to range from 0.44 to 7.5 kPa, depending on the specific tissue region and experimental conditions.^45–47^ The observed differences between the tensile strengths of our PLLA and PCL membranes and the elastic modulus of native lung tissue reflect variations in their mechanical properties.^48^ These discrepancies can be attributed to differences in fabrication methods, material composition, and structural organization between synthetic membranes and native tissue.^49–51^ It is also well recognized that biomaterials exhibiting high stability *in vitro* may demonstrate altered stability *in vivo*. Notably, PCL has been reported as a reliable long-term biomaterial for pleurodesis applications, with studies showing gradual degradation over time *in vivo*, including a 17% reduction in molecular weight over 6 months. This controlled biodegradation process enables the membrane to gradually degrade and be replaced by host tissue within the biological environment, while simultaneously providing long-term structural support. Furthermore, mechanical testing has demonstrated that PCL maintains sufficient adhesion during degradation, supporting cellular interactions over extended periods as it is biologically resorbed.^52^ Yoon et al. evaluated the *in vitro* and *in vivo* biodegradation of PLLA, demonstrating that PLLA implants retained mechanical integrity for up to 180 days in both laboratory settings and a rat model. Consistent with previous reports, the biodegradation of PLLA became apparent after approximately eight months, underscoring its advantage as a stable scaffold material for short- and medium-term biomedical applications. These findings support the suitability of PLLA as a biomaterial for long-term implants, tissue engineering scaffolds, and surgical meshes. However, comprehensive long-term follow-up studies are necessary to fully characterize its complete degradation profile and behavior in physiological environments.^53,54^ Despite these variations, our findings underscore the potential of PLLA and PCL membranes as biomimetic scaffolds for tissue engineering, although further optimization may be required to more closely replicate the mechanical properties of native tissue. The mechanical behavior of electrospun membranes is strongly influenced by fiber structural characteristics, including fiber diameter, the distribution of amorphous and crystalline phases, and fiber orientation, all of which significantly affect material performance.^19^ Previous studies have extensively documented the relationship between these structural features and mechanical properties. For example, a detailed investigation into the physical properties of polymer nanofibers demonstrated that PLLA nanofibers with diameters below 350 nm exhibited an elastic modulus of 1.0 ± 0.2 GPa.^20^ This observation highlights the complex relationship between nanofiber dimensions and mechanical performance, emphasizing the critical role of structural parameters in determining material behavior. Another study employing electrospinning to fabricate PCL membranes reported a Young’s modulus of 9 ± 1.3 MPa for the resulting scaffolds.^18^ This finding contributes to the growing body of evidence detailing the mechanical properties of electrospun membranes, underscoring their versatility and tunability for diverse applications.

In tissue engineering, understanding and controlling scaffold degradation is essential to ensure adequate structural support and to effectively guide tissue regeneration.^55,56^ It has been demonstrated that *in vivo* degradation typically occurs at a faster rate than *in vitro*, highlighting the necessity of evaluating degradation kinetics under both conditions.^57^ Importantly, the literature indicates that optimal structural integrity is maintained for at least 4 to 6 months under *in vitro* conditions at 37 °C.^58^

The degradation behavior of PLLA and PCL nanofiber membranes was monitored over six months in Ringer’s solution. After this period, PLLA and PCL membranes exhibited degradation rates of 0.8% and 1.5%, respectively (Figure 2A)**.** These results indicate that both membranes maintained substantial structural integrity without significant degradation during the six-month time frame. Notably, this exceptional stability over six months highlights the potential suitability of PLLA and PCL membranes for long-term *in vitro* airway modeling studies. Collectively, these findings suggest that PLLA and PCL membranes represent promising biomaterials for tissue engineering applications aimed at repairing complex airway structures such as the trachea and bronchi over extended durations.^59^

**Figure 2:**
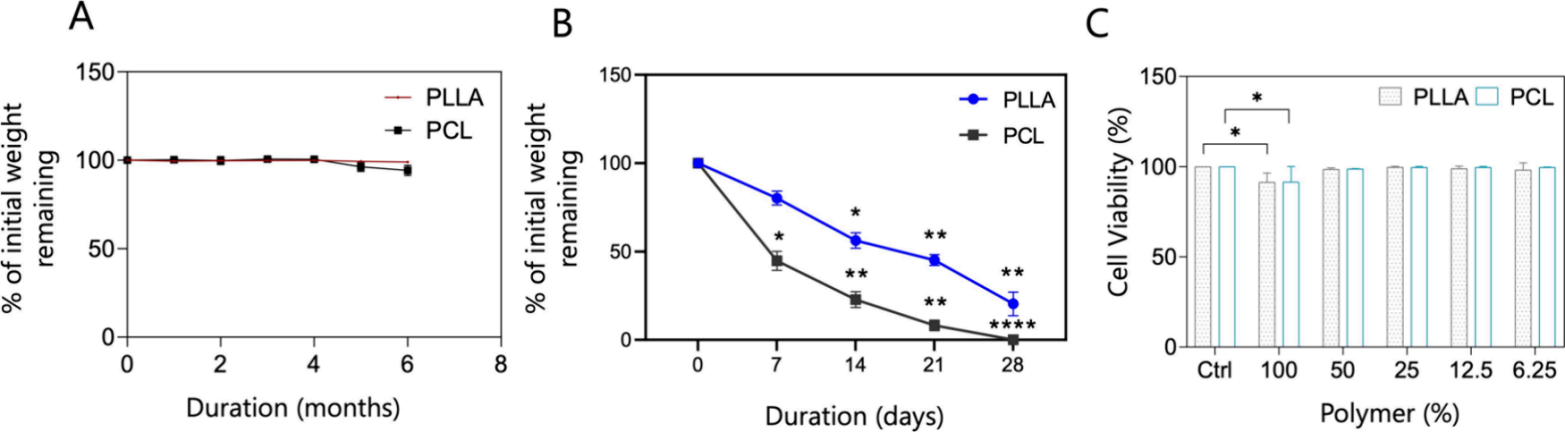
Evaluation of PLLA and PCL nanofiber membrane degradation in Ringer’s solution. Weight change of PLLA and PCL nanofiber membranes during 6 months (A). Weight change of PLLA and PCL nanofiber membranes during enzymatic degradation in proteinase K solution over 28 days (B). Cytotoxicity result of L929 stimulated with different concentrations of PLLA and PCL nanofiber membrane extract (C). ANOVA was performed to compare groups, followed by the Bonferroni post hoc test. The significance is represented by *­(*p* < 0.05), **­(*p* < 0.01), and ****­(*p* < 0.0001). The experiments were carried out at least three times in triplicate.

In addition to evaluating mechanical properties and degradation rates, assessing cytotoxicity is crucial for tissue engineering applications. Due to their favorable properties, PLLA and PCL polymers have been extensively utilized in this field.^60,61^ In the present study, the *in vitro* cytotoxicity of electrospun PLLA and PCL membranes was assessed using L929 mouse fibroblast cells. The results indicated that both PLLA and PCL membranes exhibited minimal cytotoxic effects, with cell viability remaining around 91% even at the highest tested concentration (Figure 2C). These findings align with previous reports demonstrating that PLLA and PCL membranes do not induce cytotoxicity *in vitro* and may further promote cell proliferation.^62–65^

In addition to static degradation studies, enzymatic degradation assays with Proteinase K demonstrated that PLLA and PCL membranes are susceptible to proteolytic breakdown (Figure 2B). This enzymatic sensitivity is a desirable characteristic in the context of tissue engineering, as it suggests that the membranes can be gradually degraded and replaced by host tissue *in vivo*. Notably, our 21-day ALI culture period, which was consistent with established models in the literature for achieving functional epithelial differentiation, also provided a relevant time frame to observe cell-mediated effects on membrane degradation.^66–68^ Previous studies have shown that long-term coculture with epithelial or fibroblast cells leads to progressive structural changes in biodegradable membranes due to the secretion of proteolytic enzymes and other cellular byproducts. For instance, fibroblast cultures have been reported to reduce the mechanical integrity of PLLA over time, while epithelial cell-derived proteases were shown to accelerate degradation of PCL surfaces.^69–71^ The findings indicate that cellular activity plays a significant role in the degradation kinetics of biodegradable scaffolds under physiologically relevant conditions. Future work should further explore degradation kinetics under dynamic, cell-secreting conditions to mimic physiological remodeling processes.^72–74^

To further investigate the long-term biocompatibility and functionality of PLLA and PCL nanofiber membranes, a Transwell-like insert system was utilized—an established *in vitro* cell culture platform that replicates the ALI of the lung and enables the study of cellular behavior under physiologically relevant conditions. Cell proliferation on PLLA and PCL membranes was assessed by quantifying DNA content (ng/mL), serving as an indicator of viable cell growth. Additionally, cytocompatibility with human-derived cells was evaluated using BEAS-2B bronchial epithelial cells, a well-characterized cell line frequently employed in toxicology and biomaterials research.^75,76^ Given the adherent nature of BEAS-2B cells, the impact of PLLA and PCL membranes on cell viability was evaluated by assessing their capacity to support sustained cell culture through direct cell–material interactions. This approach provided valuable insights into cell proliferation dynamics and the biocompatibility of the scaffolds under air–liquid interface conditions.

Cell culture experiments demonstrating the integration of nanofiber membranes into insert systems are presented in Figure 3A and described in detail in the Supporting Information. To assess cell adhesion and proliferation, BEAS-2B bronchial epithelial cells were cultured on PLLA and PCL membranes for 4 days, during which a 51% increase in DNA content was observed, indicating active cell proliferation (Figure 3B). To evaluate the biocompatibility of the membranes individually, cell adhesion and spreading were analyzed using SEM. SEM images acquired after 21 days of culture revealed that cells adhered along the nanofiber architecture and formed well-defined clusters on both PLLA and PCL membranes (Figure 3C,D)**.** H&E staining confirmed effective cell adhesion and proliferation on the ALI surface of the membranes. Cryosectioned and stained samples demonstrated that the bronchial epithelial cells remained viable and successfully proliferated, forming stratified epithelial structures on both PLLA and PCL membranes (Figure 3E,F).

**Figure 3:**
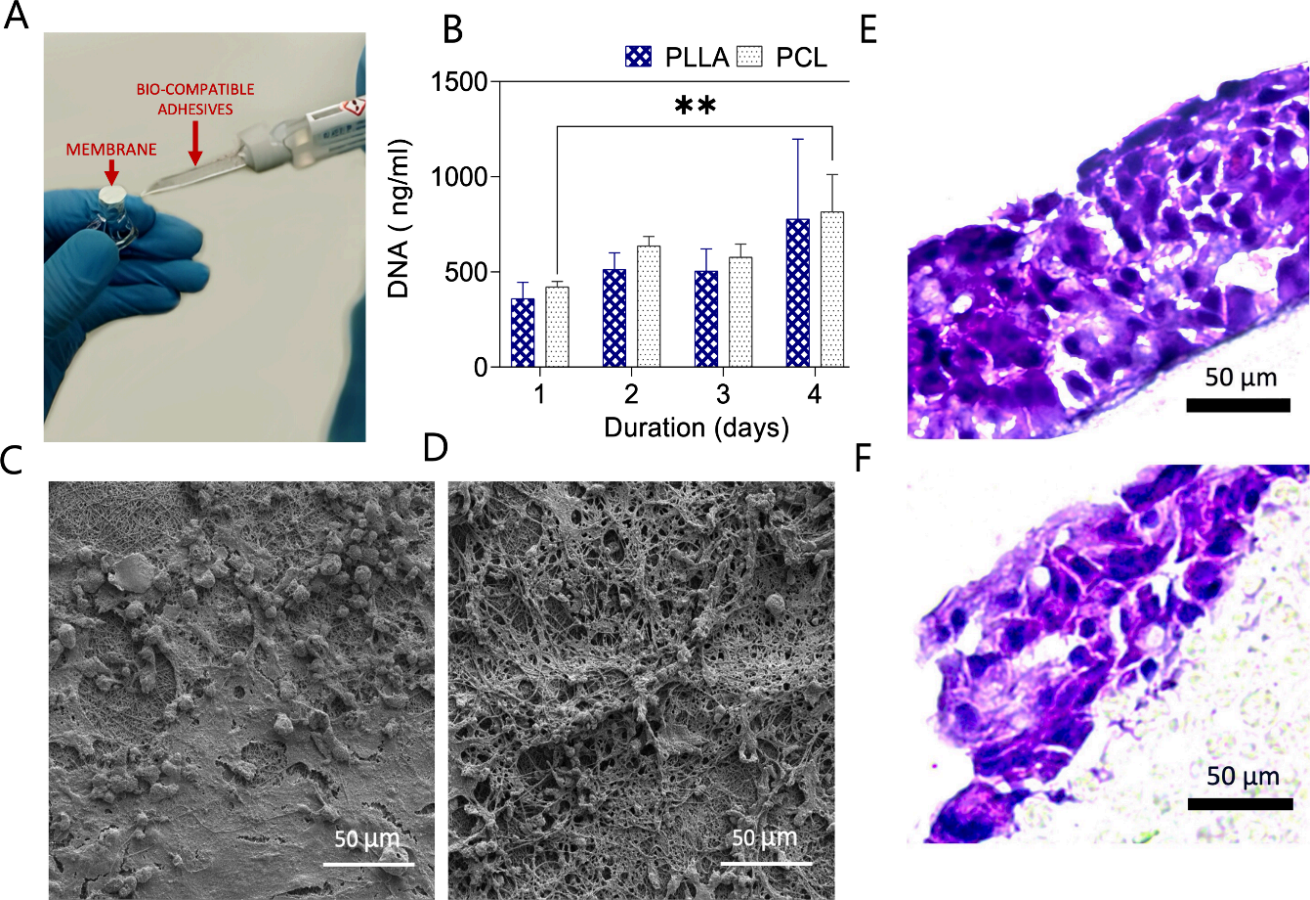
(A) Schematic representation of the experimental setup and fabrication of PLLA and PCL nanofiber membrane inserts. (B) Proliferation of BEAS-2B epithelial cells cultured on PLLA and PCL nanofiber membranes over 4 days, measured by the DNA content (ng/mL). (C, D) SEM images showing cell morphology and adhesion of epithelial cells cultured on PLLA (C) and PCL (D) nanofiber membranes after 21 days. (E, F) H&E staining of cryosectioned PLLA (E) and PCL (F) membranes, indicating successful cell attachment, proliferation, and stratification. Scale bars = 50 μm. Statistical analysis was performed using ANOVA followed by a Bonferroni post hoc test. Significance levels: *­(*p* < 0.05), **­(*p* < 0.01), ***­(*p* < 0.0001). Experiments were conducted in triplicate and repeated at least three times.

Epithelial cell proliferation on PLLA and PCL membranes was monitored during the initial 4 days following cell seeding under ALI culture conditions. Upon reaching confluency, the airway epithelial cells were exposed to the air–liquid interface to induce differentiation—a process known to arrest proliferation and initiate the expression of differentiation-specific proteins.^77–79^ Throughout the culture period, a progressive increase in cell numbers was observed, reflecting the nontoxic nature and growth-supportive properties of both membranes. These findings provide strong evidence for the biocompatibility of PLLA and PCL membranes and highlight their potential for *in vivo* applications aimed at facilitating tissue regeneration. By offering structural support and effectively mimicking the extracellular matrix, these nanofiber membranes may enhance epithelial repair and regeneration. Following the confirmation of their regenerative and biocompatible features, it is critical to validate epithelial cell adhesion and differentiation on these scaffolds. For this purpose, immunocytochemical analysis combined with SEM remains the gold standard in assessing scaffold–cell interactions.^28,80,81^ This approach facilitates the detailed visualization and characterization of cellular interactions with PLLA and PCL scaffolds, offering critical insights into their applicability for tissue engineering. To independently assess the cellular compatibility of the electrospun membranes, cell adhesion and spreading were evaluated through H&E staining and SEM. SEM images of BEAS-2B cells cultured on PLLA and PCL membranes under ALI conditions revealed spheroid-like morphologies on both membrane surfaces, indicative of successful cellular adherence.^18^ These findings demonstrate that the nanofibrous architecture promotes strong cell–cell and cell–substrate interactions, fostering a conducive environment for epithelial growth. Moreover, H&E staining supported these observations by revealing a uniform and widespread cellular distribution across the membrane surfaces, further confirming the biocompatibility of the scaffolds. Collectively, these results emphasize the critical role of comprehensive characterization methods in evaluating scaffold performance and reinforce the potential of PLLA and PCL nanofiber membranes in supporting essential cellular functions necessary for tissue regeneration.

To assess epithelial cell polarization and long-term stability on the scaffolds, we measured TEER and evaluated the expression of β-tubulin, a well-established marker of ciliated epithelial cells. TEER is a widely used quantitative indicator of tight junction integrity and epithelial barrier function in *in vitro* culture systems that simulate mucosal surfaces.^75^ In the PLLA membrane group, TEER values exhibited a steady increase from day 0 to day 21, indicating progressive barrier maturation. In the PCL membrane group, TEER measurements were recorded as 201 Ω·cm^2^ on day 0, increased to 235 Ω·cm^2^ on day 7, and subsequently declined to 201 Ω·cm^2^ and 191 Ω·cm^2^ on days 14 and 21, respectively. These findings reflect temporal variations in epithelial barrier development on different scaffold types and provide important insights into scaffold-specific effects on epithelial polarization and monolayer integrity. A regular and progressive increase in TEER values was observed in epithelial cells cultured on PLLA membranes throughout the 21 days (Figure 4A). This consistent rise in TEER was observed exclusively in the PLLA membrane group, indicating the successful establishment and maintenance of a tight epithelial barrier. This indicates that PLLA membranes effectively facilitated the establishment and sustained maintenance of a cellular barrier over 21 days, demonstrating their capability to support long-term cellular homeostasis.

**Figure 4:**
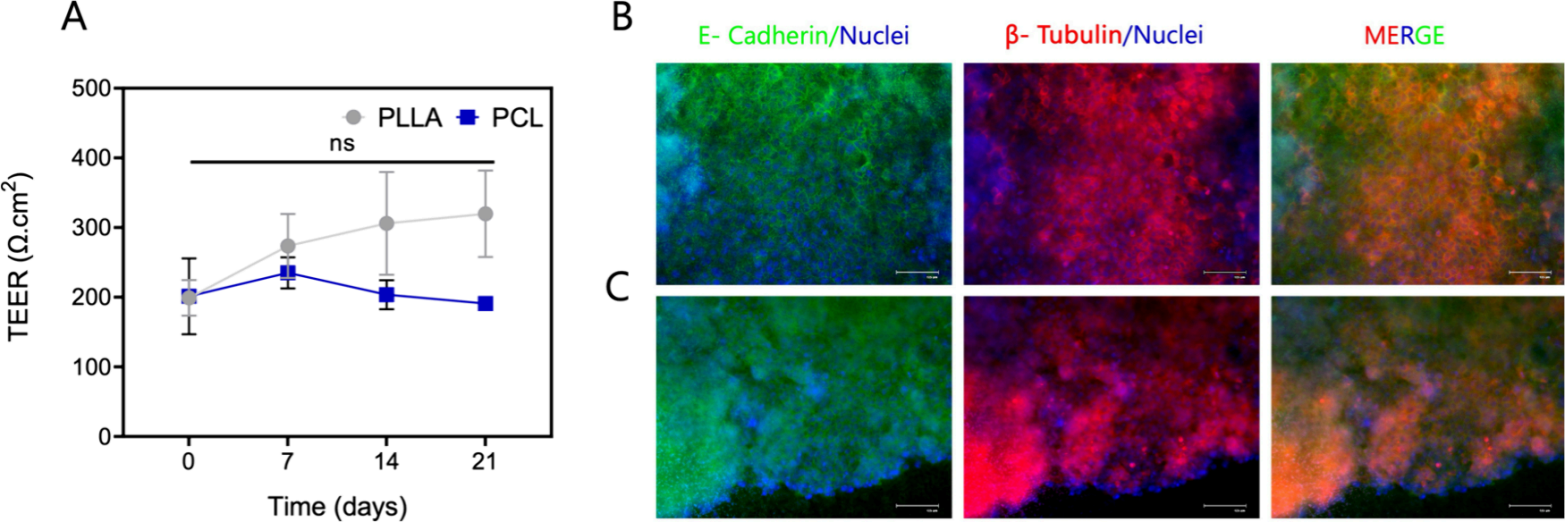
Evaluation of TEER values for epithelial cells cultured on the PLLA and PCL nanofiber membranes over 21 days of culture (A). The immunofluorescence staining of E-cadherin and β-tubulin produced by epithelial cells cultured in PLLA (B) and PCL (C) nanofiber membranes for 21 days. There was no significant difference among the groups.

Although an upward trend in TEER values was observed over time in the PLLA membrane group, the relatively high variability prevented statistical confirmation of a consistent increase. This variability likely stems from experimental fluctuations during membrane-insert handling or subtle differences in cell culture conditions. Despite this variability, the peak TEER values observed for PLLA membranes (∼250 Ω·cm^2^) are in line with or slightly exceed those previously reported for BEAS-2B cells in ALI culture. For instance, in a study evaluating bronchial epithelial cell models for asthma research, BEAS-2B cells cultured on standard 0.4 μm polyester membranes reached maximum TEER values of 100–150 Ω·cm^2^ by day 14.^31,82^ This comparison supports the functional relevance and barrier-forming potential of PLLA membranes for airway modeling applications.

In our study, BEAS-2B cells cultured on PCL membranes demonstrated attachment and β-tubulin synthesis, indicating partial differentiation. However, TEER values remained relatively stable throughout the 21-day ALI culture, suggesting limited tight junction maturation or epithelial polarization. A similar temporal trend was observed in the study by Choi et al. (2024), where TEER values increased by day 7 in 6-layer PCL meshes (18.75 ± 1.92 μm), followed by a decline at days 14 and 21. Although their study reported a recovery in TEER by day 28, our early stage results closely align with the pattern seen up to day 21, despite differences in cell type (BEAS-2B vs NHBE), membrane production, and absence of ECM coatings or dynamic culture enhancements. Structurally, our electrospun PCL membranes (144 ± 16 μm, pore size ∼0.40 μm) fall between the 6-layer (18.75 μm) and 80-layer (256.83 μm) PCL meshes used in Choi et al.’s study. These intermediate thicknesses were deliberately selected to maintain mechanical stability while avoiding diffusion limitations that could arise with overly thick scaffolds in ALI systems. Literature indicates that while thinner membranes may promote earlier barrier formation, they are often less stable over time; conversely, thicker membranes may delay barrier formation but offer sustained structural support.^83^

In contrast to the relatively flat TEER profile of PCL membranes, PLLA membranes (77 ± 12 μm) exhibited a steady increase in TEER over the 21-day period, indicating progressive barrier maturation.^83^ This difference may stem from intrinsic material properties: PLLA possesses a more favorable surface chemistry and stiffness for epithelial organization, whereas PCL is more hydrophobic and less bioactive. Moreover, differences in porosity and surface topography may have affected cell distribution and polarization on PCL membranes, contributing to reduced TEER.^84–86^ Overall, the combination of cell type, membrane architecture, and lack of biochemical cues likely limited the barrier function observed on PCL membranes. In future studies, surface modifications or ECM-based enhancements will be considered to improve epithelial compatibility and functional performance.

Immunocytochemistry analyses were conducted to verify protein expression on the membranes following confirmation of bronchial epithelial cell adhesion on PLLA and PCL membranes using the ALI culture method (Figure 4B,C). Immunostaining revealed robust E-cadherin and β-tubulin expression in differentiated bronchial epithelial cells cultured on both PLLA and PCL membranes (Figure 4B,C). Notably, microtubule protein expression serves as a critical marker of normal epithelial function.^87,88^

The current study focused on validating the structural, mechanical, and biological performance of electrospun PLLA and PCL membranes in supporting epithelial cell growth and initial differentiation at the ALI. While β-tubulin expression provides evidence of ciliated cell development and TEER measurements, along with E-cadherin expression, indicate functional barrier formation and tight junction integrity, a more comprehensive characterization of epithelial phenotypes—including goblet cells (MUC5AC), club cells (SCGB1A1), and basal cells (KRT5)—will be essential in future studies. These markers will help confirm the full pseudostratified mucociliary phenotype and further validate the regenerative potential of biodegradable membranes. Nevertheless, the current findings establish a strong proof-of-concept for using PLLA and PCL membranes as physiologically relevant scaffolds in airway tissue engineering and ALI-based disease models.^89,90^

While our study primarily focuses on the comparative performance of PLLA and PCL membranes, we acknowledge the absence of direct experimental comparisons with commercially available PET-based membranes. Nevertheless, previous studies have highlighted key limitations of PET membranes in ALI culture systems. Notably, widely used commercial PET and PC membranes fail to adequately mimic the stiffness and mechanical properties of the lung extracellular matrix.^91^ Although a direct quantitative comparison with commercial PET inserts was not conducted in this study, our findings are consistent with existing literature that emphasizes the superior performance of PLLA and PCL membranes in supporting epithelial differentiation and maintaining barrier function.^92^

PLA membranes demonstrate significant potential for pulmonary applications owing to their inherent biodegradability and excellent biocompatibility.^93,94^ PLA membranes exhibit promising potential for pulmonary drug delivery applications. However, despite these advantages, challenges persist, such as insufficient mechanical strength and limited control over pore architecture.^95^ Ongoing research is dedicated to improving the properties of PLA membranes by employing advanced fabrication techniques such as electrospinning and phase separation to optimize their functionality for tissue engineering, drug delivery, and other biomedical applications.^95^ PCL membranes have demonstrated significant potential in a range of biomedical applications, including lung-on-chip devices and pleurodesis treatments. In particular, composite membranes combining PCL with collagen exhibit enhanced biocompatibility and show promise for integration into microfluidic lung models, thereby advancing *in vitro* respiratory system research.^96^ However, despite these promising findings, limitations remain in the current body of research. More comprehensive clinical investigations are required to thoroughly elucidate the full potential and limitations of PCL-based materials across diverse medical applications.

The extant literature supports the hypothesis that PLLA and PCL-based electrospun Transwell membranes are promising biomaterial candidates for long-term epithelial cell culture, with potential applications in both *in vitro* modeling and *in vivo* regenerative therapies. The high porosity, adjustable fiber architecture, and mechanical stability of these materials have been demonstrated to promote epithelial cell adhesion, proliferation, and polarization. Although both PLLA and PCL electrospun membranes were capable of supporting airway epithelial cell attachment, E-cadherin and β-tubulin expression under ALI conditions, distinct differences in functional performance were observed. PLLA membranes exhibited a progressive increase in TEER values over time, indicating the formation of a tight and polarized epithelial barrier. This may be attributed to their moderate stiffness (6.82 ± 0.57 MPa), lower thickness (77 ± 12 μm), and more favorable surface characteristics that facilitate tight junction maturation and barrier integrity.

In contrast, PCL membranes—despite showing initial cell adhesion and structural protein expression—displayed a gradual decline in TEER values. This may result from their greater thickness (144 ± 16 μm), slightly lower elasticity (5.60 ± 1.54 MPa), and possible differences in oxygen/nutrient diffusion or surface-cell interactions. These factors could hinder the maintenance of a uniform and functional monolayer, leading to reduced paracellular resistance. Therefore, while both materials are biocompatible, PLLA appears more suitable as a long-term ALI model substrate, offering improved support for epithelial polarization and barrier function. Further optimization of PCL membranes, such as surface functionalization or composite strategies, may be required to enhance their performance in such applications. Furthermore, the biodegradable nature of both polymers allows for gradual scaffold resorption and natural tissue integration. The microporous structure of these materials has been shown to support epithelial growth, while also permitting mesenchymal infiltration and extracellular matrix deposition. This property renders them particularly well-suited for *in vivo* applications in diseases characterized by epithelial barrier dysfunction, including asthma, COPD and pulmonary fibrosis.^70,93^

While this study focused on the epithelial compatibility and scaffold performance of PLLA and PCL membranes, future work will involve coculture models integrating fibroblasts, endothelial cells, or immune components to investigate epithelial–mesenchymal and epithelial–immune crosstalk. These efforts will be essential to further validate the potential of these biodegradable membranes for advanced lung tissue engineering applications.

## 4 Conclusions

This study provides valuable insights into the potential of electrospun nanofiber membranes for lung tissue regeneration within the field of tissue engineering. Through comprehensive characterization—including structural analysis, mechanical testing, degradation profiling, and cytotoxicity assessment—PLLA and PCL membranes emerged as feasible alternatives for lung tissue engineering applications. Notably, these electrospun membranes demonstrate advantageous features that closely mimic the native lung extracellular matrix, such as bead-free fiber morphology, uniform fiber distribution, and an optimal pore size conducive to cell attachment and function. Current airway 3D culture applications predominantly utilize air–liquid interface (ALI) culture systems based on nonbiodegradable membranes. These systems are primarily designed for molecular research and drug discovery related to respiratory diseases, yet they fall short in addressing clinical challenges such as bronchial tissue injury. The replacement of conventional nonbiodegradable membranes in Transwell inserts with biodegradable alternatives, coupled with their integration into ALI culture platforms, offers significant advantages. This approach allows for the fabrication of membranes with tailored structures suitable for potential *in vivo* applications, thereby more accurately recapitulating the native living tissue environment.

The use of FDA-approved biocompatible and tunable membranes holds significant promise for facilitating long-term ALI cultures, potentially allowing epithelial cells to develop functional structures such as cilia, tight junctions, and mucus that closely resemble *in vivo* conditions. However, it is important to note that our current study provides initial, short-term evidence, and further long-term investigations are necessary to confirm the development of these complex epithelial features experimentally. Moreover, such membranes may offer solutions to clinical challenges, including tracheal defects and bronchial injury, by reducing tissue damage. They could serve as effective scaffolds in graft development, acting as ‘submucosal tissue analogs’ to support tissue regeneration. Future studies will be essential to validate these applications and to optimize membrane properties for clinical translation.

## Supplementary Material



## Data Availability

All data supporting the findings of this study are included within the paper and its Supporting Information file. Raw data are available upon reasonable request.

## Notes

Cytotoxicity analysis, assembling of nanofiber membranes on inserts, trans-epithelial electrical resistance (TEER), last stage of the tensile strength test, and mechanical properties of electrospun PLLA and PCL nanofibers (PDF)

## #

Department of Neurosciences, Institute of Health Sciences, Izmir Kâtip Celebi University, İzmir 35620, Türkiye (S.S.C.)

## $

Division of Pharmacology, Utrecht Institute for Pharmaceutical Sciences, Faculty of Science, Utrecht University, Utrecht 3508 TB, The Netherlands (H.A.B.).
