# Supplementary material for: Biodegradable Nanofiber Membranes for Air–Liquid Interface Culture: Advancing Airway In Vitro Models
Source: ACS Omega. 2025 Aug 21;10(35):39693–705. doi: 10.1021/acsomega.5c03198 (PMC12423884; doi:10.1021/acsomega.5c03198)
Supplement: Supplementary file 1 [file ao5c03198_si_001.pdf]

Supporting Information for:

Biodegradable Nanofiber Membranes for Air-Liquid Interface Culture: Advancing Airway In Vitro Models

Sema Tuncer[a], Secil Subasi Can[a #], Hayriye Akel Bilgic[b $], Busra Kilic[b], Gulcin Gunal[c], Halil Murat Aydin[a], Cagatay Karaaslan[a,b*]

[a]Hacettepe University, Institute of Science and Centre of Bioengineering, Bioengineering Division, 06800, Cankaya, Ankara, Türkiye

[b]Hacettepe University, Faculty of Science, Department of Biology, Molecular Biology Section, 06800 Cankaya, Ankara, Türkiye

[c]Akdeniz University, Faculty of Medicine, Department of Plastic Surgery, 07070, Campus, Konyaaltı, Antalya, Türkiye

* E-mail: cagatayk@hacettepe.edu.tr

*Corresponding Author: Cagatay KARAASLAN

Supplementary Methods

1. Cytotoxicity Analysis

The investigation of the *in vitro* cytotoxic effects of electrospun PLLA and PCL membranes was conducted in accordance with the EN ISO 10993-5 Cytotoxicity Tests and the EN ISO 10993-12 Biological Evaluation of Medical Devices - Sample Preparation and Reference Materials standards [1].

The membranes were cut into 2 cm x 6 cm dimensions according to the extraction rate measurements specified in the standard **Table 2**. Then, they were sterilized with 70% (v/v) alcohol for 2 hours. Then, to prepare the extraction medium, serum-free medium was added to the membranes and incubated at 37°C in a CO_2 incubator for 72 hours. After 72 hours, the membranes were removed, and serum and antibiotics were added to the extraction medium.

L929 mouse fibroblast cells (NCTC clone 929, ATCC) were used in cytotoxicity test of membranes. The cells were sub-cultured in flasks with DMEM low glucose medium (Catalog no: 12-707F, Lonza) containing 10% (v/v) FBS (Biowest, France), 1% (v/v) P/S (PS-B, Capricorn, Germany), and 1% (v/v) L-glutamine (Sigma G6392). L929 cells were detached from the surface using trypsin (0.01%)/EDTA (10 mM) and resuspended in a medium before cell seeding. The cells that were multiplied in 75 cm^2 flasks at 37°C in an incubator containing 95% CO_2 and 5% humidity and reached 80-90% growth density were passaged and seeded into 96-well culture dishes with $1x10^4$ cells in each well. After the cells were planted in the 96-well medium and cultured for 24 hours, they were treated with different concentrations of the extraction medium (100%, 50%, 25%, 12.5%, 6.25%) (100 µL medium/well) **(Figure 2C).** 10 µL of 3-(4,5-dimethylthiazol-2-yl)-diphenyltetrazolium bromide (MTT) (5 mg/mL in PBS) solution was added to all wells and allowed to incubate. After the incubation at 37°C for 3 h, the medium was removed and 100 µL DMSO was added to each well. The absorbance was read at 570 nm on the Multimode Plate Reader (Perkin Elmer, MA, USA) and plotted as percent viability values. The experiments were carried out in triplicate, and the medium in which the cells grew was used as a negative control, and DMSO was used as a positive control [1, 2].

2. *Assembling of Nanofiber Membranes on Inserts*

The polyethylene terephthalate (PET) membranes on Corning transwells were excised using a scalpel to prepare biodegradable transwell inserts. Membrane-free transwell inserts were sterilized in 70% (v/v) ethanol for 6 h. At the end of the period, the inserts were washed with PBS and left to dry. For this purpose, PLLA and PCL membranes were cut into round discs with a diameter of approximately 7 mm. The non-biodegradable PET membranes in the existing transwells were replaced with biodegradable nanofiber membranes and glued with Kwik-Sil (WPI, Sarasota, FL), which is a biocompatible adhesive (silicone elastomer) **(Figure 3A).** The membranes adhered to the transwells were subjected to sterilization with 70% (v/v) ethanol and then washed with PBS and then dried [3]**.** All procedures were carried out in Class II, Type B2 Biological Safety Cabinet (ESCO Micro Pte. Ltd., Singapore).

3. *Trans-Epithelial Electrical Resistance (TEER)*

After the PLLA and PCL membranes were attached to the transwell, they were placed in a 24-well plate first covered with FBS and kept overnight in an incubator at 37 ^oC with 95% CO_2 and 5% humidity for the serum proteins to precipitate on the membrane surface and for better adhesion of the membranes. The next day, the epithelial cells covering the flask were trypsinized and removed, and the cells were seeded apically onto the membrane surfaces at a rate of 1.0×10^5 cells/membrane and placed in the incubator. Then, the standard ALI culture procedure was applied. The cells, which were fed with 500 µL of BEGM medium from the basal and 100 µL from the apical for 3 days, were taken to differentiation at the end of the 3rd day. At this stage, the liquid in the upper chamber was entirely removed and the apical part of cells was directly exposed to air, and the cells were fed only from the basal part. TEER measurements were performed on days 1, 7, 14 and 21 during the 21-day differentiation period [4]**.**

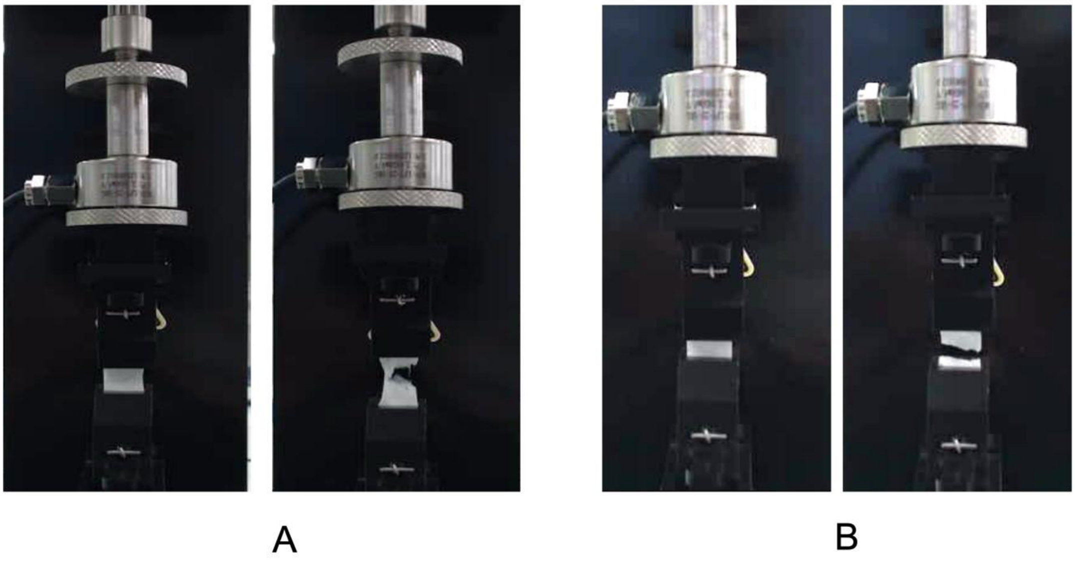

Figure S1. The last stage of the tensile strength test of electrospun PLLA **(A)** and PCL **(B)** nanofibers.

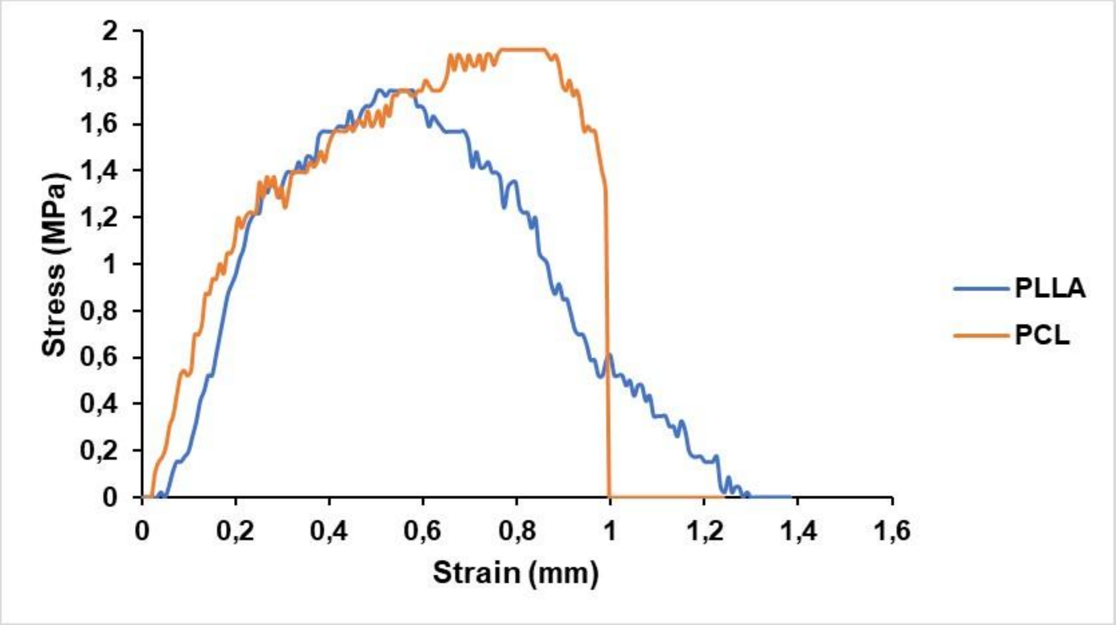

Figure S2. Mechanical properties of electrospun PLLA and PCL nanofibers.

Present Addresses:

S.S.C: Izmir Kâtip Celebi University, Institute of Health Sciences, Department of Neurosciences, 35620, İzmir, Türkiye

$ H.A.B: Utrecht University, Division of Pharmacology, Utrecht Institute for Pharmaceutical Sciences, Faculty of Science, 3508 TB, Utrecht, The Netherlands

References

(1) EN ISO 10993-5"Biological Evaluation of Medical Devices- Part 5: Tests for In Vitro Cytotoxicity. EN ISO 10993-12 "Biological Evaluation of Medical Devices-Part 12: Sample Preparation and Reference Materials".

(2) Herrero-Herrero, M.; Alberdi-Torres, S.; González-Fernández, M. L.; Vilarino-Feltrer, G.; Rodríguez-Hernández, J. C.; Vallés-Lluch, A.; Villar-Suárez, V. Influence of chemistry and fiber diameter of electrospun PLA, PCL and their blend membranes, intended as cell supports, on their biological behavior. *Polymer Testing* **2021**, *103*, 107364.

(3) Mahoney, C.; Conklin, D.; Waterman, J.; Sankar, J.; Bhattarai, N. Electrospun nanofibers of poly (ε-caprolactone)/depolymerized chitosan for respiratory tissue engineering applications. *Journal of Biomaterials Science, Polymer Edition* **2016**, *27* (7), 611-625.

(4) Morris, G. E.; Bridge, J. C.; Brace, L. A.; Knox, A. J.; Aylott, J. W.; Brightling, C. E.; Ghaemmaghami, A. M.; Rose, F. R. A novel electrospun biphasic scaffold provides optimal three-dimensional topography for in vitro co-culture of airway epithelial and fibroblast cells. *Biofabrication* **2014**, *6* (3), 035014.
